# Caffeine Mitigates Lung Inflammation Induced by Ischemia-Reperfusion of Lower Limbs in Rats

**DOI:** 10.1155/2015/361638

**Published:** 2015-11-16

**Authors:** Wei-Chi Chou, Ming-Chang Kao, Chung-Tai Yue, Pei-Shan Tsai, Chun-Jen Huang

**Affiliations:** ^1^Department of Anesthesiology, Taipei Tzu Chi Hospital, Buddhist Tzu Chi Medical Foundation, 289 Jianguo Road, Xindian District, New Taipei City 23142, Taiwan; ^2^School of Medicine, Tzu Chi University, No. 701, Sec. 3, Zhongyang Road, Hualien 97004, Taiwan; ^3^Department of Pathology, Taipei Tzu Chi Hospital, Buddhist Tzu Chi Medical Foundation, 289 Jianguo Road, Xindian District, New Taipei City 23142, Taiwan; ^4^Graduate Institute of Nursing, College of Nursing, Taipei Medical University, 250 Wushing Street, Taipei 11042, Taiwan

## Abstract

Reperfusion of ischemic limbs can induce inflammation and subsequently cause acute lung injury. Caffeine, a widely used psychostimulant, possesses potent anti-inflammatory capacity. We elucidated whether caffeine can mitigate lung inflammation caused by ischemia-reperfusion (IR) of the lower limbs. Adult male Sprague-Dawley rats were randomly allocated to receive IR, IR plus caffeine (IR + Caf group), sham-operation (Sham), or sham plus caffeine (*n* = 12 in each group). To induce IR, lower limbs were bilaterally tied by rubber bands high around each thigh for 3 hours followed by reperfusion for 3 hours. Caffeine (50 mg/kg, intraperitoneal injection) was administered immediately after reperfusion. Our histological assay data revealed characteristics of severe lung inflammation in the IR group and mild to moderate characteristic of lung inflammation in the IR + Caf group. Total cells number and protein concentration in bronchoalveolar lavage fluid of the IR group were significantly higher than those of the IR + Caf group (*P* < 0.001 and *P* = 0.008, resp.). Similarly, pulmonary concentrations of inflammatory mediators (tumor necrosis factor-*α*, interleukin-1*β*, and macrophage inflammatory protein-2) and pulmonary myeloperoxidase activity of the IR group were significantly higher than those of the IR + Caf group (all *P* < 0.05). These data clearly demonstrate that caffeine could mitigate lung inflammation induced by ischemia-reperfusion of the lower limbs.

## 1. Introduction

Lower limb ischemia can be caused by a variety of clinical conditions, including critical limb ischemia, abdominal aortic aneurysm, and traumatic arterial injury [1–3]. Therapies that can restore perfusion to the ischemic limb(s) are performed to reduce injury caused by ischemia. However, reperfusion of the ischemic limb(s) can in turn induce inflammation and cause remote organ injury [4–6]. In this regard, the lung is one of the organs most vulnerable to remote injury subsequent to ischemia-reperfusion [2, 4, 6].

Caffeine (1,3,7-trimethylxanthine) is a widely used psychostimulant. Caffeine alone is used clinically in the treatment of headache, respiratory depression in neonates, obesity, and postprandial hypotension [7]. These abovementioned effects of caffeine are mediated by inhibition of methylxanthine-sensitive adenosine receptors [7]. In addition, caffeine has been shown to possess potent anti-inflammatory capacity [8, 9]. Using animal models, previous studies confirmed that caffeine (especially high dose caffeine) exerted significant therapeutic effects against traumatic brain injury [10] and oleic oil-induced lung injury [11]. Caffeine was also shown to exert protective effects against myocardial ischemia-reperfusion [12].

To date, the question of whether caffeine could be protective of lung tissues against the adverse effects of ischemia-reperfusion of the lower limbs remains unstudied. To elucidate further, we thus conducted this study. This systematic study used an established anesthetized rodent model of ischemia-reperfusion of the lower limbs [4, 13] to determine if systemic application of caffeine at reperfusion would mitigate anatomical and biochemical markers of lung inflammation and pathology.

## 2. Materials and Methods

This animal study was approved by the Institutional Animal Use and Care Committee of Taipei Tzu Chi Hospital, Buddhist Tzu Chi Medical Foundation (102-IACUC-014). Rats were treated according to National Institutes of Health guidelines. A total of 48 adult male Sprague-Dawley rats (200 g to 250 g; BioLASCO Taiwan Co., Ltd., Taipei, Taiwan) were used in this study. All rats were fed a standard laboratory chow and water at liberty until the experimental day.

### 2.1. Animal Preparation

All rats were anesthetized with an intraperitoneal (ip) injection of a mixture of zoletil (40 mg/kg; Virbac, Carros, France) and xylazine (Rompun TS, Bayer, Leverkusen, Germany) and were placed in a supine position. The right carotid artery was cannulated with a polyethylene (PE-50) catheter for continuous hemodynamic monitoring and blood drawing. A tracheostomy was performed and a 16-gauge angiocatheter was inserted as a tracheostomy tube. Blood pressure and respiratory rate were continuously monitored throughout the experiments. Supplemental doses of zoletil/xylazine mixture (13/3 mg/kg, ip) were given every 30–60 minutes until the end of the study to ensure and maintain adequate anesthesia.

### 2.2. Experimental Protocols

The protocol of lower limb ischemia-reperfusion injury was modified from previously published reports [4, 13]. In brief, bilateral lower limb ischemia-reperfusion was performed by applying rubber band tourniquets high around each thigh for 3 hours followed by reperfusion for 3 hours. Half of the rats received the lower limb ischemia-reperfusion injury protocol. To control for the effects of manipulations, the remaining rats received a sham-operation, that is, anesthesia, carotid artery cannulation, and tracheostomy, but no introduction of the rubber band tourniquets and limb ischemia-reperfusion.

### 2.3. Experimental Groups

All rats were randomly assigned to one of the four experimental groups (*n* = 12 in each group): the sham-operation (Sham), the sham plus caffeine (Sham + Caf), the lower limbs ischemia-reperfusion (IR), and the IR plus caffeine (IR + Caf) groups. Rats of the Sham + Caf and the IR + Caf groups received caffeine (50 mg/kg, ip; Sigma-Aldrich, St. Louis, MO, USA) immediately after reperfusion. The dose of caffeine (50 mg/kg ip) was chosen according to a previous study demonstrating that caffeine at this dose could mitigate oleic acid-induced lung injury in mice [11]. To control for the effects of treatment vehicle, rats of the Sham and the IR group received normal saline (1.0 mL, ip) at comparable time point. After 3 hours of reperfusion, all rats were euthanized with neck dislocation.

### 2.4. Lung Tissue Collection and Bronchoalveolar Lavage

Thoracotomy was performed to facilitate lung tissue harvesting. The left main bronchus was tied and the left lungs were removed. The left lung tissues were snap-frozen in liquid nitrogen and stored at −80°C for subsequent analysis. To facilitate histological analysis, the right lung tissues of six rats from each group were infused with 4% formaldehyde through the tracheostomy tube and then removed. To facilitate bronchoalveolar lavage fluid (BALF) analysis, the right lungs of the other six rats from each group were lavaged five times with 3 mL sterile normal saline, as we have previously reported [13, 14]. The BALF was then collected. To maximize the efficacy of BALF collection, suction was performed twice after each lavage. The five fractions of BALF from each rat were pooled together and saved for the subsequent analysis.

### 2.5. Histological Analysis

The formaldehyde-infused lung tissues were embedded in paraffin wax, serially sectioned, and then stained with hematoxylin and eosin. Lung tissue inflammation was evaluated using a light microscope by a pathologist who was blind to this study. Histologic characteristics, including edematous change of the alveolar wall, hemorrhage, vascular congestion, and polymorphonuclear leukocytes (PMN) infiltration, were used to evaluate lung inflammation, according to our previous report [14].

### 2.6. Total Cells Number and Protein Concentration in BALF

An aliquot of the pooled BALF (50 *μ*L) from each rat was diluted 1 : 1 with trypan blue dye (Life Technologies, Grand Island, NY, USA) and the total cells number was counted using a standard hemocytometer, using our previously reported protocol [13, 14]. The remaining pooled BALF from each rat was centrifuged (3000 rpm for 5 minutes at 15°C) and then the supernatants were collected. The protein concentration of the BALF supernatant was analyzed using a BCA protein assay kit (Pierce Biotechnology, Inc., Rockford, IL, USA), as directed by the manufacturer's protocol. The BALF samples were analyzed in triplicate.

### 2.7. Inflammatory Mediators and Myeloperoxidase (MPO) Activity

The frozen lung tissues were processed according to our previous reports [13, 14]. For inflammatory mediators, frozen lung tissues were weighed and homogenized with a tissue homogenizer (MICCRA D-1, ART Prozess & Labortechnik GmbH & Co. KG, Müllheim, Germany) in 5 volumes of RIPA buffer (150 mM NaCl, 1% NP-40, 0.5% sodium deoxycholate, 0.1% sodium dodecyl sulfate, and 50 mM Tris-HCl, pH 7.5; all chemicals were from Sigma-Aldrich) and incubated at 4°C in RIPA buffer. Following centrifugation (14,000 rpm at 4°C for 20 minutes), the tissue supernatants were collected. After measuring the protein concentration using a BCA protein assay kit (Pierce), the concentrations of cytokines (e.g., tumor necrosis factor-*α* (TNF-*α*) and interleukin-1*β* (IL-1*β*)) and chemokine (e.g., macrophage inflammatory protein-2, MIP-2) in the tissue supernatants were analyzed in triplicate using commercial enzyme-linked immunosorbent assay (ELISA) kits (ELISA kits for TNF-*α* and IL-1*β*, Pierce; MIP-2 ELISA kit; R&D Systems, Inc., Minneapolis, MN, USA). ELISA was performed as per the manufacturers' protocols.

Pulmonary MPO activity from snap-frozen tissue was quantified, as per our previous reports [13, 14], to measure the activity of the infiltrated PMN, an indicator of lung inflammation [13]. Lung tissue samples were weighed and homogenized for 1 minute in 15 volumes of PE buffer (0.01 M KH_2_PO_4_ with 1 mM EDTA). Following homogenization and centrifugation (14,000 rpm at 4°C for 20 minutes), the pellets were collected and resuspended in 15 volumes of cetyltrimethylammonium bromide buffer (13.7 mM) with acetic acid (50 mM). The resuspended pellets were then sonicated and centrifuged (10,000 rpm for 15 minutes at 15°C). The supernatants were collected and incubated in a water bath for 2 hours at 60°C. MPO activity was measured using a MPO fluorometric detection kit (Enzo Life Science, Plymouth Meeting, PA, USA), according to the manufacturer's instructions. The samples were analyzed in triplicate. All chemicals were from Sigma-Aldrich.

### 2.8. Malondialdehyde (MDA) Assay

Snap-frozen lung tissue homogenates were assayed for MDA using thiobarbituric acid test, as per our previous published protocols [13, 14], to quantify the status of lipid peroxidation [15]. In brief, snap-frozen lung tissues were weighed and homogenized in 5 volumes of RIPA buffer on ice. After centrifugation (2000 rpm at 4°C for 10 minutes), the supernatants were collected and stored on ice. The MDA concentrations of the supernatants were measured using a commercial MDA assay kit (TBARS assay kit, Cayman Chemical Co., Ann Arbor, MI, USA), according to the manufacturer's instruction. The samples were also analyzed in triplicate.

### 2.9. Statistical Analysis

One-way analysis of variance with the Bonferroni-Dunn test was used for multiple comparisons. Data were presented as mean ± standard deviation. The significance level was set at 0.05. A commercial software package (SigmaStat for Windows, SPSS Science, Chicago, IL) was used for data analysis.

## 3. Results

### 3.1. Lung Histology Data

**Figure 1:**
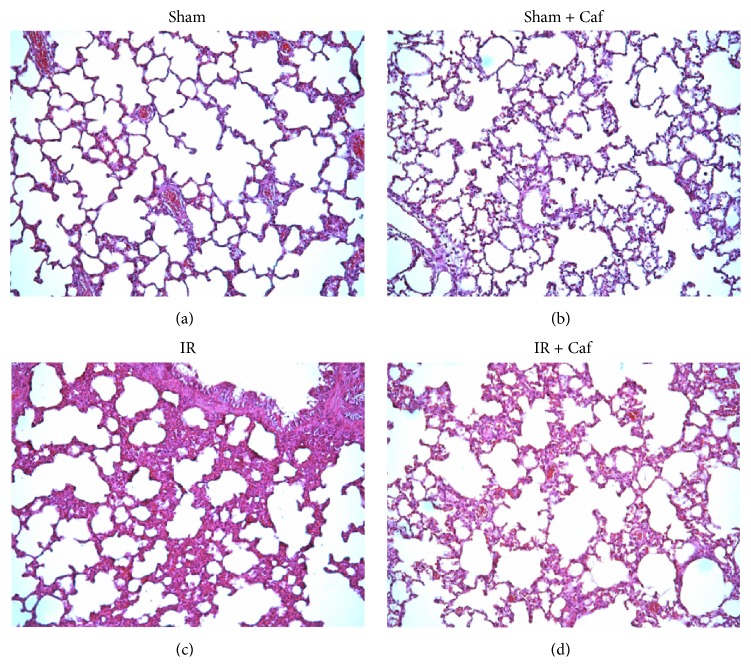
Representative microscopic findings of the lung tissues stained with hemotoxylin & eosin (100x). (a) The sham-operation (Sham) group revealed normal to mild lung inflammation characteristics. (b) The sham plus caffeine (Sham + Caf) group revealed normal to mild lung inflammation characteristics. (c) The lower limb ischemia-reperfusion (IR) group revealed severe lung inflammation characteristics. (d) The IR plus caffeine (IR + Caf) group revealed mild to moderate lung inflammation characteristics. Rats of the Sham + Caf and the IR + Caf groups received caffeine (50 mg/kg, intraperitoneal injection) immediately after reperfusion. To control the effects of vehicle, rats of the Sham and the IR group received normal saline (1.0 mL, intraperitoneal injection) at comparable time point.

Histological analysis revealed normal to mild lung inflammation characteristics in the Sham and the Sham + Caf groups (Figures 1(a) and 1(b)). The lung tissues of the IR group revealed severe inflammation characteristics (Figure 1(c)). Moreover, the lung tissues of the IR + Caf group revealed mild to moderate lung inflammation characteristics (Figure 1(d)).

### 3.2. BALF Data

**Figure 2:**
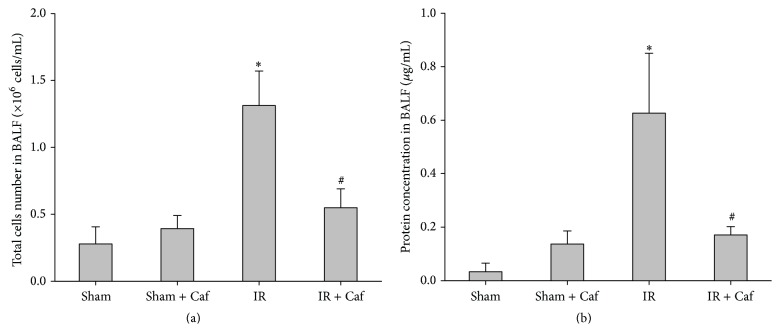
Total cells number (a) and protein concentration (b) in bronchoalveolar lavage fluid (BALF). Sham: the sham-operation group. Sham + Caf: the sham plus caffeine group. IR: the lower limb ischemia-reperfusion group. IR + Caf: the IR plus caffeine group. Rats of the Sham + Caf and the IR + Caf groups received caffeine (50 mg/kg, intraperitoneal injection) immediately after reperfusion. To control for the effects of the treatment vehicle, rats of the Sham and the IR group received normal saline (1.0 mL, intraperitoneal injection) at the comparable time point. One-way analysis of variance with the Bonferroni-Dunn test was used for multiple comparisons. The significance level was set at 0.05. Data were derived from 6 rats from each group and presented as mean ± standard deviation. ^*∗*^ *P* < 0.05: the IR group versus the Sham group. ^#^ *P* < 0.05: the IR + Caf group versus the IR group.

In BALF from the Sham group, the total cells number was 0.28 ± 0.13 × 10^6^ cells/mL and the protein concentration was 0.03 ± 0.03 *μ*g/mL (Figures 2(a) and 2(b)). The total cells number (0.39 ± 0.09 × 10^6^ cells/mL) and protein concentration (0.14 ± 0.05 *μ*g/mL) in BALF of the Sham + Caf groups were similar to those of the Sham group (Figures 2(a) and 2(b)). The total cells number (1.31 ± 0.26 × 10^6^ cells/mL) and protein concentration (0.63 ± 0.22 *μ*g/mL) of the IR group were significantly higher than those of the Sham group (*P* < 0.001 and =0.002, resp.; Figures 2(a) and 2(b)). Moreover, the total cells number (0.55 ± 0.14 × 10^6^ cells/mL) and protein concentration (0.17 ± 0.03 *μ*g/mL) of the IR + Caf group were significantly lower than those of the IR group (*P* < 0.001 and *P* = 0.008, resp.; Figures 2(a) and 2(b)).

### 3.3. Pulmonary Inflammatory Mediators and MPO Activity Data

**Figure 3:**
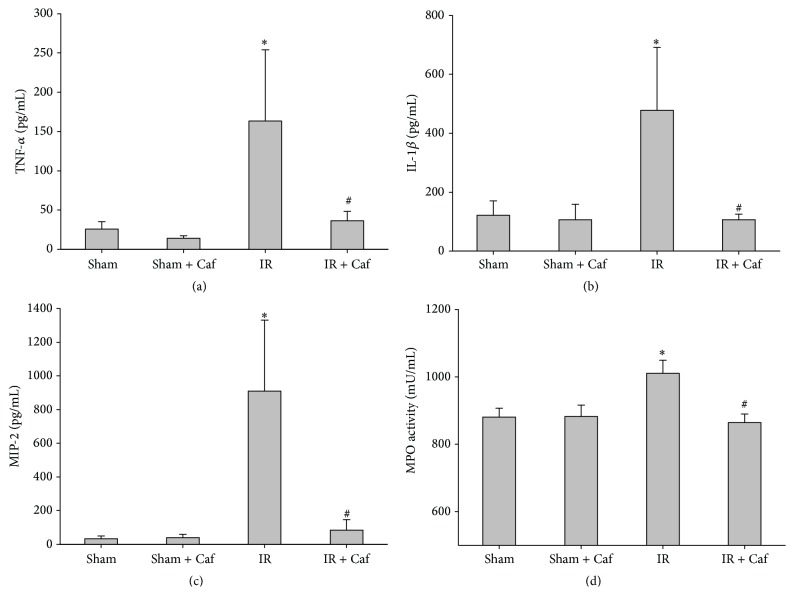
The pulmonary concentrations of (a) tumor necrosis factor-*α* (TNF-*α*), (b) interleukin-1*β* (IL-1*β*), (c) macrophage inflammatory protein-2 (MIP-2), and (d) myeloperoxidase (MPO) activity. Sham: the sham-operation group. Sham + Caf: the sham plus caffeine group. IR: the lower limb ischemia-reperfusion group. IR + Caf: the IR plus caffeine group. Rats of the Sham + Caf and the IR + Caf groups received caffeine (50 mg/kg, intraperitoneal injection) immediately after reperfusion. To control for the effects of treatment vehicle, rats of the Sham and the IR group received normal saline (1.0 mL, intraperitoneal injection) at the comparable time point. One-way analysis of variance with the Bonferroni-Dunn test was used for multiple comparisons. The significance level was set at 0.05. Data were derived from 6 rats from each group and presented as mean ± standard deviation. ^*∗*^ *P* < 0.05: the IR group versus the Sham group. ^#^ *P* < 0.05: the IR + Caf group versus the IR group.

In the Sham group, the pulmonary concentration of TNF-*α* was 25.8 ± 9.4 pg/mL, IL-1*β* was 121.8 ± 48.7 pg/mL, MIP-2 was 31.7 ± 17.2 pg/mL, and MPO activity was 880.7 ± 26.2 mU/mL (Figures 3(a)–3(d)). The pulmonary concentrations of TNF-*α* (14.0 ± 3.2 pg/mL), IL-1*β* (106.2 ± 53.5 pg/mL), and MIP-2 (38.9 ± 20.0 pg/mL) as well as the pulmonary MPO activity (882.8 ± 33.4 mU/mL) of the Sham + Caf group were similar to those of the Sham group (Figures 3(a)–3(d)). The pulmonary concentrations of TNF-*α* (163.5 ± 90.4 pg/mL), IL-1*β* (478.4 ± 213.3 pg/mL), and MIP-2 (909.3 ± 422.5 pg/mL) as well as the pulmonary MPO activity (1010.7 ± 38.8 mU/mL) of the IR group were significantly higher than those of the Sham group (all *P* < 0.001; Figures 3(a)–3(d)). In contrast, the pulmonary concentrations of TNF-*α* (36.4 ± 12.1 pg/mL), IL-1*β* (106.5 ± 38.4 pg/mL), and MIP-2 (83.2 ± 62.2 pg/mL) as well as the pulmonary MPO activity (864.7 ± 25.4 mU/mL) of the IR + Caf group were significantly lower than those of the IR group (all *P* < 0.001; Figures 3(a)–3(d)).

### 3.4. Pulmonary MDA Data

**Figure 4:**
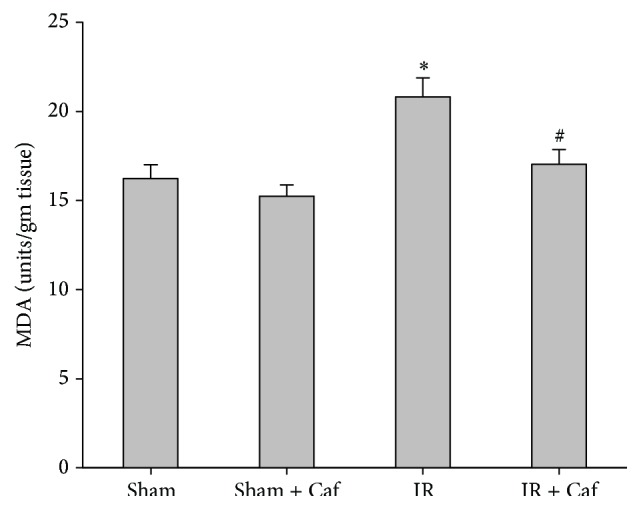
The pulmonary malondialdehyde (MDA) concentrations. Sham: the sham-operation group. Sham + Caf: the sham plus caffeine group. IR: the lower limb ischemia-reperfusion group. IR + Caf: the IR plus caffeine group. Rats of the Sham + Caf and the IR + Caf groups received caffeine (50 mg/kg, intraperitoneal injection) immediately after reperfusion. To control for the effects of treatment vehicle, rats of the Sham and the IR group received normal saline (1.0 mL, intra-peritoneal injection) at the comparable time point. One-way analysis of variance with the Bonferroni-Dunn test was used for multiple comparisons. The significance level was set at 0.05. Data were derived from 6 rats from each group and presented as mean ± standard deviation. ^*∗*^ *P* < 0.05: the IR group versus the Sham group. ^#^ *P* < 0.05: the IR + Caf group versus the IR group.

The pulmonary MDA concentration of the Sham group was 16.2 ± 0.8 units/gm tissue and the Sham + Caf group was 15.2 ± 0.6 units/gm tissue (Figure 4). The pulmonary MDA concentration (20.8 ± 1.1 units/gm tissue) of the IR group was significantly higher than that of the Sham group (*P* < 0.001; Figure 4). In contrast, the pulmonary MDA concentration (17.0 ± 0.8 units/gm tissue) of the IR + Caf group was significantly lower than that of the IR group (*P* < 0.001; Figure 4).

## 4. Discussion

The results of this study are consistent with previous studies [2, 4, 6] and confirmed that ischemia-reperfusion of the lower limbs can induce significant inflammation of the lung. Furthermore, the present study clearly demonstrates that in this rodent model caffeine mitigates lung inflammation induced by lower limbs ischemia-reperfusion although the underlying mechanisms were not investigated.

It is well established that inflammation is a crucial mechanism in mediating remote organ injury induced by ischemia-reperfusion of the lower limbs [4, 13] but a mechanist appraisal of the protective effect of caffeine can, at this stage, only be alluded to indirectly from an extensive literature of related studies. Expression of inflammatory mediators is tightly regulated by the crucial upstream transcriptional factor nuclear factor-*κ*B (NF-*κ*B) [16]. Pervious data also indicated that ischemia-reperfusion can activate NF-*κ*B expression [17]. As such, it is plausible that the protective, anti-inflammatory effects of caffeine in the lung may act, in part, through inhibition of NF-*κ*B activation. This concept is supported by previous data that caffeine could inhibit NF-*κ*B activation in endotoxin-stimulated microglia [18]. Interestingly, poly(ADP-ribose) polymerase- (PARP-) 1 is a cofactor for NF-*κ*B mediated upregulation of inflammatory mediators [19]. However, in cultured epithelial and endothelial cells, caffeine metabolites, at physiological levels, inhibit PARP-1 [20]. In addition, cyclic AMP (cAMP) is a potent inhibitor of NF-*κ*B [21]. Degradation of cAMP is tightly regulated by phosphodiesterases and inhibition of phosphodiesterases can increase the level of cAMP which in turn inhibits NF-*κ*B activity [22]. Of relevance is the fact that caffeine is a nonselective phosphodiesterase inhibitor [23]. In light of the cited literature, we speculate that the mechanisms underlying the anti-inflammatory role of caffeine in ischemia-reperfusion of the lower limbs may also involve its effects on inhibiting PARP-1 and/or phosphodiesterases.

In addition to inflammation, oxidative stress also plays a crucial role in mediating the development of lung injury induced by ischemia-reperfusion of the lower limb [4, 13, 24, 25]. Previous studies demonstrated that ischemia-reperfusion of the lower limb significantly increased xanthine oxidase activity and promoted oxidant generation as well as lipid peroxidation while the application of antioxidants mitigated acute lung injury in this model [13, 24, 25]. A considerable body of evidence documents the direct free radical scavenging capacity of caffeine [26] while it has also been recognized that caffeine has the ability to enhance the expression of the upstream transcription factor nuclear factor-E2-related factor 2 (Nrf2) and the downstream antioxidant enzyme system, including superoxide dismutase (SOD) and catalase [27]. In our study, levels of pulmonary MDA, an indicator of lipid peroxidation, were significantly increased by ischemia-reperfusion of the lower limb and this increase was significantly attenuated by the application of caffeine, a result consistent with an antioxidant action of caffeine. As such, our results suggest that a component of the protective effects of caffeine may be derived from its actions on the pathways mediating oxidative stress.

In this rodent study, the protective effects of caffeine are clear. However, the question of whether the therapeutic effects of caffeine are dose-dependent remains unstudied. As previously mentioned, the dose of caffeine employed in this study was based upon the protective effect of 50 mg/kg caffeine (ip) on acute lung injury induced by oleic acid in mice [11]. Somewhat paradoxically in the same study, two lower doses of caffeine, 5 and 15 mg/kg, aggravated the lung injury induced by oleic acid [11]. Moreover, the therapeutic potential of low doses of caffeine, that is, 5 or 15 mg/kg, had also been investigated using a rodent model of traumatic brain injury [10], but these doses failed to modulate indices of traumatic brain injury [10]. Although the present study employed only a single high dose of caffeine, these inconsistent literature findings with respect to dose and ischemic model prompted us to perform a series of preliminary studies to test the effects of lower doses of caffeine (i.e., 10 or 25 mg/kg) on the modulation of the upregulation of pulmonary TNF-*α* induced by lower limb ischemia-reperfusion. Our preliminary data revealed that low doses of caffeine (10 or 25 mg/kg) exerted no significant modulation of the upregulation of pulmonary TNF-*α* in lower limbs ischemia-reperfusion (data not shown). Though more studies are needed before further conclusions can be made, nevertheless, these data clearly indicate that the significant anti-inflammatory effects of caffeine can only be observed with high dose.

To elucidate further, we are currently conducting a follow-up study to compare the therapeutic potentials between 100 mg/kg caffeine and 50 mg/kg caffeine using the same model. The preliminary data obtained from the follow-up study revealed that 100 mg/kg caffeine could significantly inhibit the increases in total cells number and protein concentration in BLAF induced by lower limb ischemia-reperfusion in rats (please see Supplemental Figure 1 in Supplementary Material available online at http://dx.doi.org/10.1155/2015/361638). However, our preliminary data also revealed that total cells number and protein concentration in BLAF in rats receiving lower limb ischemia-reperfusion plus 100 mg/kg caffeine were not significantly different from those in rats receiving lower limb ischemia-reperfusion plus 50 mg/kg caffeine (Supplemental Figure 1). These data seem to suggest that the therapeutic potentials of 100 mg/kg caffeine and 50 mg/kg caffeine were similar. In line with this notion, we thus speculate that there may be a ceiling effect regarding the therapeutic potential of high dose caffeine in mitigating lung inflammation induced by lower limb ischemia-reperfusion. If so, then this observation will definitively limit the clinical application of caffeine in this regard. More studies are needed before further conclusion can be reached.

It should be noted that this study confirmed that caffeine exerted significant anti-inflammatory effects in the early phase of ischemia-reperfusion (i.e., within 6 hours). However, the question of whether caffeine can produce prolonged effects against lower limbs ischemia-reperfusion remains unstudied.

## 5. Conclusions

Caffeine mitigates lung inflammation induced by lower limbs ischemia-reperfusion in rats.

## Supplementary Material

Supplmental Figure 1 illustrates the preliminary data of total cell number (A) and protein concentration (B) in bronchoalveolar lavage fluid (BALF). Sham+N/S: the sham-operation plus normal saline group. Sham+C50: the Sham plus 50 mg/kg caffeine group. Sham+C100: the Sham plus 100 mg/kg caffeine group. IR+N/S: the lower limb ischemia-reperfusion plus normal saline group. IR+C50: the IR plus 50 mg/kg caffeine group. IR+C100: the IR plus 100 mg/kg caffeine group. Rats of the Sham+C50, the Sham+C100, the IR+C50 and the I/R+C100 groups received caffeine (intra-peritoneal injection) immediately after reperfusion. To control for the effects of treatment vehicle, rats of the Sham+N/S and the IR+N/S group received normal saline (1.0 mL, intra-peritoneal injection) at the comparable time point. One way analysis of variance with the Bonferroni-Dunn test was used for multiple comparisons. The significance level was set at 0.05. Data were derived from 3 rats from each group and presented as mean ± standard deviation. ∗P<0.05 the IR+N/S group versus the Sham+N/S group. #P<0.05 the IR+C50 group or the I/R+C100 group versus the IR+N/S group.
