# Supplementary material for: Caffeine Mitigates Lung Inflammation Induced by Ischemia-Reperfusion of Lower Limbs in Rats
Source: Mediators Inflamm. 2015 Nov 16;2015:361638. doi: 10.1155/2015/361638 (PMC4663348; doi:10.1155/2015/361638)
Supplement: Supplementary file 1 — Supplmental Figure 1 illustrates the preliminary data of total cell number (A) and protein concentration (B) in bronchoalveolar lavage fluid (BALF). Sham+N/S: the sham-operation plus normal saline group. Sham+C50: the Sham plus 50 mg/kg caffeine group. Sham+C100: the Sham plus 100 mg/kg caffeine group. IR+N/S: the lower limb ischemia-reperfusion plus normal saline group. IR+C50: the IR plus 50 mg/kg caffeine group. IR+C100: the IR plus 100 mg/kg caffeine group. Rats of the Sham+C50, the Sham+C100, the IR+C50 and the I/R+C100 groups received caffeine (intra-peritoneal injection) immediately after reperfusion. To control for the effects of treatment vehicle, rats of the Sham+N/S and the IR+N/S group received normal saline (1.0 mL, intra-peritoneal injection) at the comparable time point. One way analysis of variance with the Bonferroni-Dunn test was used for multiple comparisons. The significance level was set at 0.05. Data were derived from 3 rats from each group and presented as mean ± standard deviation. ∗P<0.05 the IR+N/S group versus the Sham+N/S group. #P<0.05 the IR+C50 group or the I/R+C100 group versus the IR+N/S group. [file 361638.f1.pdf]

Supplemental Figure 1

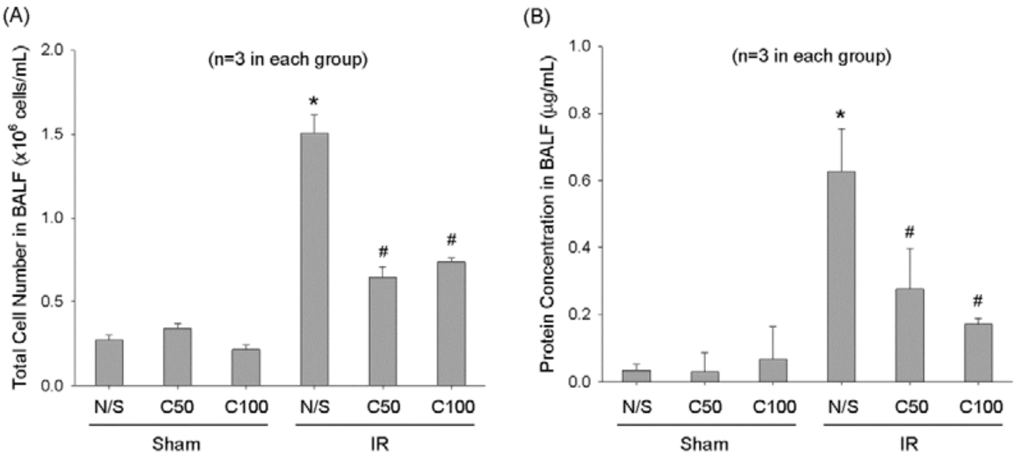

Supplmental Figure 1. The preliminary data of total cell number (A) and protein concentration (B) in bronchoalveolar lavage fluid (BALF). Sham+N/S: the sham-operation plus normal saline group. Sham+C50: the Sham plus 50 mg/kg caffeine group. Sham+C100: the Sham plus 100 mg/kg caffeine group. IR+N/S: the lower limb ischemia-reperfusion plus normal saline group. IR+C50: the IR plus 50 mg/kg caffeine group. IR+C100: the IR plus 100 mg/kg caffeine group. Rats of the Sham+C50, the Sham+C100, the IR+C50 and the I/R+C100 groups received caffeine (intra-peritoneal injection) immediately after reperfusion. To control for the effects of treatment vehicle, rats of the Sham+N/S and the IR+N/S group received normal saline (1.0 mL, intra-peritoneal injection) at the comparable time point. One way analysis of variance with the Bonferroni-Dunn test was used for multiple comparisons. The significance level was set at 0.05. Data were derived from 3 rats from each group and presented as mean±standard deviation. *P<0.05 the IR+N/S group *versus* the Sham+N/S group. #P<0.05 the IR+C50 group or the I/R+C100 group *versus* the IR+N/S group.
